# Clinical characteristics and dynamics of disability progression in a cohort of patients with multiple sclerosis in Latvians

**DOI:** 10.1007/s10072-024-07404-z

**Published:** 2024-02-23

**Authors:** Jolanta Kalnina, Ilva Trapina, Nikolajs Sjakste, Natalia Paramonova

**Affiliations:** 1 Genomics and Bioinformatics, Institute of Biology of the University of Latvia, Riga, LV-1004 Latvia; 2 Department of Medical Biochemistry of the University of Latvia, Riga, LV-1004 Latvia

**Keywords:** Multiple sclerosis, Disease progression, EDSS score, Relapsing–remitting MS, Secondary progressive MS

## Abstract

There is wide variation in the time from the onset to secondary progressive multiple sclerosis (MS) and some controversy regarding the clinical characteristics of the courses (phenotypes) of MS. The present study aimed to characterize demographic and clinical factors that potentially influence long-term disability progression in the cohort of Latvian MS patients. A descriptive longitudinal incidence study was conducted using a cohort of 288 MS patients beginning in 2011 (disease duration from 1 to 51 years). Socio-demographic and clinical information from the first visit to 15/20 years was analysed in groups stratified by gender and visits at five-time points (the first visit; after a year or 2; after 5 ± 1 year; after 10 ± 2 years; after 15–20 years). Our study was dominated by patients from urban areas and non-smokers. The female/male ratio was 2.4:1; the distribution of clinical courses at the first visit was consistent with most European studies. The most common symptom at presentation in our study was optic manifestations, followed by sensory disturbances and motor deficits. In the Latvian study, gender was not a significant influencing factor on the rate of disease progression; however, patient age was statistically significantly associated with EDSS (Expanded Disability Status Scale) value at the first visit. Early clinical features of MS are important in predicting the disability accumulation of patients. Despite the small differences regarding the first MS symptoms, the disability outcomes in the cohort of Latvian patients are similar to other regions of the world.

**Supplementary Information:**

The online version contains supplementary material available at 10.1007/s10072-024-07404-z.

## Introduction

Multiple sclerosis (MS) is a chronic, inflammatory, demyelinating, neurodegenerative disease of the central nervous system with an unpredictable clinical course and highly variable clinical manifestations [1].

The neurological disorders associated with MS usually affect the most productive years of life, most often in young and middle-aged individuals [1, 2]. The worldwide prevalence of this disease has increased significantly, increasing by 10% every 5 years over the past three decades, and is diagnosed in approximately 2.8 million people worldwide in 2020 (35.9 per 100′000 population) [3], of which more than 700,000 are in Europe [4]. The prevalence of MS in Europe varies widely (from 30 to 227 cases per 100,000 population) [4]. According to studies, together with other Baltic states, Latvia remains a region with a higher MS incidence rate compared to other nations [5], with 2300 confirmed MS cases in a country with about 1.89 million inhabitants (ranging from 122 per 100,000 population) [https://datareportal.com/reports/digital-2023-latvia]. A limited set of studies has been conducted on the manifestations and clinical course of multiple sclerosis in the Baltic region.

Three main clinical courses (phenotypes) of MS have been identified: relapsing–remitting MS (RRMS), secondary-progressive MS (SPMS), and primary-progressive MS (PPMS). Since there are no objective criteria for dividing clinical phenotypes, Lublin and the authors proposed to classify them based on disease activity (active/inactive forms considering recurrence rate and findings on MRI) and disease progression (progressive/non-progressive forms based on a more objective measurement – EDSS (Expanded Disability Status Scale)) [2, 6, 7]. Worldwide, most patients with MS (approximately 85%) are initially diagnosed with RRMS, and within 15 to 20 years, 50–60% of these patients develop SPMS [8–10]. This suggests that both RRMS and SPMS may be part of a single disease continuum. There is wide variation in the time from the onset to the progression of SPMS [11] and some controversy regarding the clinical characteristics (initial symptoms, MRI features, disease prognosis, etc.) of both RRMS and SPMS clinical courses (phenotypes) of MS [11, 12].

Until now, the reason why multiple sclerosis progresses rapidly in one patient and to a lesser extent than in another remains unclear. Thus, there is a need for a better understanding of the factors, both clinical and environmental, that appear to be associated with the disability process in multiple sclerosis to provide personalized patient management approaches to prevent and slow the progression of multiple sclerosis and therefore the risk of mortality.

The purpose of this study was also to address this lack of information by describing the clinical presentation and disability progression in the population of MS patients in Latvia. A set of data on MS cases collected at different time points allows close observation of the course of the disease over time and the identification of demographic and clinical factors that potentially predict the progression of MS.

## Patients and methods 

### Study design and data source

We conducted a descriptive incidence longitudinal study (2011–2020) using the clinical data from a collection of MS patients, (disease duration of 1–51 years), based on the Latvian Maritime Medicine Centre, Vecmilgravis Hospital (Riga, Latvia), according to Helsinki Declaration and approved by the Central Medical Ethics Committee of Latvia (Protocol Nr. 01–29.1/17). Written informed consent was obtained from all participants of the study. The MS collection formed for our study in 2011 includes clinical data for 288 patients, which amounted to 18.48% of the Latvian MS cohort in that period (1558 patients were registered in 2011 in Latvia) and 14.18% of the total MS cohort (2030 patients) registered in 2022.

### Outcome measurements

The following pre-selected information was gathered and assessed from the clinic database for each patient: socio-demographic information, disease progress information: year and age of first symptoms, age at diagnosis, the amount of time that elapsed between onset of symptoms and diagnosis, disease subtype, estimated Expanded Disability Status Scale (EDSS) at diagnosis and last encounter, Disease-Modifying Therapy (DMT). The frequency of autoimmune comorbidity in each patient was evaluated. The history of comorbidity was evaluated at the first visit via interview. We also obtained the history of MS in first and second-degree relatives of MS patients.

### Cohort identification and selection 

MS patients were assigned to RRMS and SPMS groups. MS diagnosis will be classified according to the Poser criteria (clinically or laboratory-defined MS; Poser et al., 1983) or 2010 Revisions to the McDonald Criteria as bout-onset MS (relapsing–remitting/secondary progressive) or chronic-onset MS (primary progressive/progressive relapsing). Diagnostic criteria include clinical and paraclinical laboratory assessments emphasizing the need to demonstrate the dissemination of lesions in space and time and to exclude alternative diagnoses. Although the diagnosis can be made on clinical grounds alone, magnetic resonance imaging of the central nervous system can support, supplement, or even replace some clinical criteria [13]. Secondary progressive multiple sclerosis is diagnosed retrospectively and involves a clinical course characterized by a progressive accumulation of neurological disability, independent of relapses, following an initial relapsing–remitting (RR) phase. We defined progressive disease according to Lublin et al. as a steadily increasing, objectively documented neurologic dysfunction or disability without unequivocal recovery, admitting fluctuations and stability phases [6], and it is based on frequent EDSS (Expanded Disability Status Scale) evaluations and suggestive of SPMS with a worsening in the EDSS step (1.0-point with EDSS ≤ 5.5 or 0.5-point with EDSS ≥ 6.0), with a minimum score of 4.0 and a pyramidal functional system score of ≥ 2 [14]. This worsening should be confirmed at least 3 months within the same functional system score that leads to progression [14]. Secondary progression denotes the continuous worsening of neurological impairment, independent of relapses, over a period of at least 6 or 12 months [6, 15].

Socio-demographic and clinical data of the period from the first visit were analysed in the MS cohort, stratified by gender, to describe the dynamics of these characteristics over time (during 5 visits at five-time points: the first visit; after a year or 2; after 5 ± 1 year; after 10 ± 2 years; after 15—20 years).

### Statistical analysis

For metric variables, mean values with standard deviations and 95% confidential interval (CI 95%), median with interquartile rank, and interval from min to max were reported. For categorical variables, distributions, frequencies, and 95% confidential interval were presented.

Normal distribution for numerical variables was determined by the Shapiro–Wilk or Kolmogorov–Smirnov test, depending on group size.

To calculate differences between groups of metric variables: age at the first symptoms, age at the beginning of the disease or at the time of diagnosis, the time interval from the first symptoms to the diagnosis, EDSS score at the beginning of the disease, and at follow-up, during the progression from RRMS to SPMS, the analysis was chosen depending on group normal distribution: for two groups *T*-test (both groups with normal distribution) or Mann–Whitney *U*-tests was used; for more than two groups ANOVAs (all groups with normal distribution) or Kruskal–Wallis tests were performed. For more than two groups post-hoc analyses were used depending on group homogeneity. The relationship between metric size and nominal size was determined using the eta value.

Pearson's chi-squared (χ^2^) test or Fisher’s exact test was performed to calculate differences in distributions of nominal variables: gender, number of other autoimmune diseases; occurrence of MS among first- and second-degree family members; family history of other autoimmune disorders, the current course of the disease, prevalent symptoms at disease onset, disease-modifying treatments. The relationship between two nominal values was determined using Kramer's V coefficient.

Paired data, or data of one patient at five-time points, were analysed using the Friedman Test since changes in time points were not in normal distributions.

## Results 

### Population characteristics

The demographic characteristics of the cohort are presented in Table 1. In 2011, the study identified 288 people diagnosed with MS, 18.5% of the 1558 registered MS patients in Latvia. For a one-way analysis with a Type I error or alpha value set at 0.05, the study was designed to run at the 80% level to reveal a true difference between groups. Thus, this number of patients was sufficient to conduct a study with high reliability and power, given that 213 patients should have been considered a representative MS collection for Latvia at that time. The collection of this study includes patients who first applied to the MS Department of the Marine Medical Centre in the period from 1985 to 2011, and thus having a disease duration from (1) one to 51 years. In the collection of 288 MS patients, 204 (71%) are women, which corresponds to an average gender ratio of 2.4:1. Most patients (about 60%) were registered in the capital of Latvia (Riga), and the remaining cases were approximately equally distributed in other cities of the country (17.7%) and in rural areas (22.6%). About half of the cohort of patients (47.64%) have higher education, and about a quarter of patients have secondary special (25.65%) and secondary education (21.99%).

**Table 1:** Descriptive statistical indices of demographic characteristics for Multiple sclerosis cohort from Latvian population

Socio-demographic indicators (number of patients with info)	Subgroup	Patients		
		*N*	%	CI 95%
Sex distribution (288)	Females Males	204 84	70.83 29.17	65.58—76.08 23.92—34.42
Distribution of residence (288)	City of the Republic City Outside the city	172 51 65	59.72 17.71 22.57	54.06—65.38 13.30—22.12 17.74—27.40
Education level (191)	Basic education Secondary education Secondary special education Higher Education	9 42 49 91	4.71 21.99 25.65 47.64	1.71—7.71 16.12—27.86 19.46—31.84 40.56—54.72
Smoking (222)	Yes Never Rejects	40 124 58	18.02 55.86 26.13	12.96—23.08 49.33—62.39 20.35—31.91

*N* number of patients; %—frequency of distributions; CI 95%—95% confidential interval of distributions

### Clinical characteristics

The clinical (nominal) characteristics of the cohort are presented in Table 2 and 3. Of the 241 cases of the disease, the presence of disability was diagnosed as prevailing in female patients 144 (84.7%) to male 53 (74.6%), however, this difference is not statistically significan*t (p* = *0.07, V* = *0.12).*

**Table 2:** Descriptive statistical indices of clinical characteristics for Multiple sclerosis cohort stratified by sex from Latvian population

Disease-related indicators (patients with info)		The whole group			Gender				Statistical analysis^	
					Female		Male			
		*N*	%	CI 95%	*N*	%	*N*	%	*p*	*V*
Disability* (241)	Yes No	197 44	81.74 18.26	76.86—86.62 13.38—23.14	144 26	84.71 15.29	53 18	74.65 25.35	0.07	0.12
A family history of illness (238)	Yes No	27 211	11.34 88.66	7.31—15.37 84.63—92.69	23 145	13.69 86.31	4 66	5.71 94.29	0.08	0.11
Other autoimmune diseases (237)	Yes No	36 201	15.19 84.81	10.62—19.76 80.24—89.38	28 140	16.67 83.33	8 61	11.59 88.41	0.32	0.06
First symptoms (282)	Visual impairment Sensory disturbances Motor deficit Polysymptomatic onset Brainstem symptoms Cerebellar symptoms Cognitive impairment	71 57 50 44 31 27 2	25.18 20.21 17.73 15.60 10.99 9.57 0.71	20.11—30.25 15.52—24.90 13.27—22.19 11.36—19.84 7.34—14.64 6.14 – 13.00 0.00—1.69	51 37 31 33 25 21 2	25.50 18.50 15.50 16.50 12.50 10.50 1.00	20 20 19 11 6 6 0	24.39 24.39 23.17 13.41 7.32 7.32 0.00	0.46	0.15
Exacerbation in 1st year after 1st symptoms (287)	No one One Two More than two	41 214 26 6	14.29 74.56 9.06 2.09	10.24—18.34 69.52—79.6 5.74—12.38 0.43—3.75	23 157 19 5	11.27 76.96 9.31 2.45	18 57 7 2	21.43 67.86 8.33 2.38	0.21	0.14
Time till 2nd exacerbation (238)	Up to 12 months Over 12 months	116 122	48.74 51.26	42.39—55.09 44.91—57.61	87 87	50.00 50.00	29 35	45.31 54.69	0.56	0.04

^—statistical analysis between gender groups; N – number of patients; %—Frequency of distributions; CI 95%—95% confidential interval of distributions; p – statistical signification; V – Cramer’s V coefficient; * in Latvia, disability is assigned to patients by the “State Commission of Physicians for the Examination of Health and Working Capacity,” which evaluates the patient’s medical history, state of health and prognosis of the disease

**Table 3:** Disease-related characteristics for Multiple sclerosis cohort in the 1st visit from Latvian population

Disease-related indicators (patients with info)	Subgroup	*N*	%
Clinical activity (267)	No	12	4.49
	Yes	255	95.51
Magnetic resonance activity (216)	No	115	53.24
	Yes	101	46.76
Disease subtype (286)	RRMS	235	82.17
	SPMS	51	17.83
Initiation of therapy (286)	No	195	68.18
	Yes	91	31.82

*N* number of patients; % frequency of distributions; *RRMS* relapsing–remitting multiple sclerosis, *SSMS* secondary-progressive multiple sclerosis

There was no significant difference between male and female patients regarding the presence of other autoimmune diseases and predominant symptoms at disease onset. Adverse autoimmune diseases were present in 15.2% of cases from the general disease group with a slight prevalence in the female group (16.7% to male 11.6%). The female group was also found to predominate to the male group in terms of the number of cases in the family history of the disease (13.7% compared to 5.7% of cases).

The most common of the 282 reported cases were visual impairment 71 (25.2%) and sensory disturbances 57 (20.2%). The motor deficit as the first symptom occurred in 18% of cases in general and prevailed in the male group of patients (23.2%) to the female group (15.5%).

Based on the analysis of cases of exacerbation of the disease in the first year after the onset of the first symptoms, only one incident was presented in 74.56% of patients from the general group; no incident was observed in 14.3% of cases, among which the male group of patients prevailed concerning the female (21.4% vs 11.27%.) The period before the second exacerbation in half of the Latvian cohort of patients (48.7%) was about 12 months (Table 2).

At the first visit, the start of drug therapy was initiated in 91 (32%) patients. Of these, the 1st line of immunomodulatory and immunosuppressive drug therapy (interferons, glatiramer acetate, teriflunomide) was in 85 cases (93%). Clinical activity of the disease was recorded in 255 (96%)) patients out of 267 examined from the total experimental group. Magnetic resonance activity was present in 101 (47%) patients out of 215 examined. In the magnetic resonance analysis, new gadolinium-enhancing or new/enlarging T2 lesions were considered. In a group of 286 patients, 235 (82%) were diagnosed with Relapsing Remitting MS and 51 (18%) were distributed with Secondary Progressive MS (Table 3).

The mean age at the disease onset in these patients was 29.40 ± 9.42 years (29.11 ± 9.27 for females and 31.1 ± 9.80 for males) (Table 4). The minimum age of first symptoms was seven (7) years and the maximum was 54 years.

**Table 4:** Clinical data (numerical) that may influence the course of the disease

Group	Indicators								Statistical analysis^	
	Mean	SD	Min	Max	CI 95%		Median	IQR	*p*	*η*
Age of first symptoms, years (289 patients)										
All	29.40	9.42	7.00	54.00	28.31	30.49	29.00	14.00	-	-
Females	29.11	9.27	7.00	54.00	27.83	30.39	29.00	14.00	0.57	0.05
Males	30.11	9.80	13.00	53.00	27.98	32.23	28.50	14.00		
RRMS stage period before transition to SPMS stage, years (130 patients)										
All	13.35	9.05	1.00	53.00	11.78	14.92	12.00	12.00	-	-
Females	13.96	9.55	2.00	53.00	11.95	15.96	12.00	13.00	0.37	0.10
Males	11.98	7.73	1.00	32.00	9.50	14.45	11.00	13.00		
Patient age RRMS > SPMS, years (130 patients)										
All	42.98	10.55	18.00	67.00	41.15	44.82	43.00	15.00	-	-
Females	42.24	10.73	18.00	65.00	40.00	44.49	42.50	14.25	0.23	0.11
Males	44.65	10.07	24.00	67.00	41.43	47.87	44.00	16.00		

*SD* standard deviation; *CI 95%* 95% confidential interval of the mean; *IQR* interquartile range

^—statistical analysis between gender groups; *p*—statistical signification of *T*-tests or Mann Whitney (underlined) tests; η (eta) – association coefficient

*RRMS* relapsing–remitting multiple sclerosis, *SSMS* secondary-progressive multiple sclerosis

**Fig. 1:**
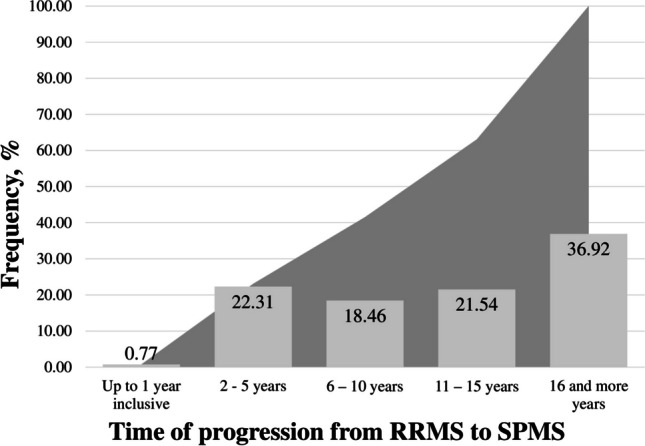
Time distribution of Multiple sclerosis progression (from relapsing–remitting (RRMS) to secondary progressive (SPMS)) in patient groups. The grey background is cumulative frequency

Of all patients, 130 progressed from RRMS to SPMS over a time of 20 years. There were no significant differences between male and female patients regarding the period of the RRMS stage before the transition to the SPMS stage (Table 4) with average disease progressing in 13.35 ± 9.05 years (13.96 ± 9.55 years for females and 11.98 ± 7.73 years for males). The approximate age of transition to the SPMS stage also turned out to be approximately the same between the sexes: 42.98 ± 10.55 years (42.24 ± 10.73 years for females and 44.65 ± 10.07 years for males). A significant proportion (36.92%) progressed in 16 or more years after the onset of the first symptoms (Fig. 1), but in more than half of the patients, disease progression takes place within 15 years, or during this time the transition from RRMS to SPMS occurs in at least 63.1%.

### Dynamics of MS-related clinical characteristics by visits

To characterize the dynamics of main MS-related clinical characteristics among the population of Latvia, their changes over time were studied by visits at five main time points: V1—1st visit; V2—Visit after one or two years from 1st visit; V3—Visit after 5 ± 1-year from 1st visit; V4—Visit after 10 ± 2 years from 1st visit; V5—Visit after 15–20 years from 1st visit.

**Fig. 2:**
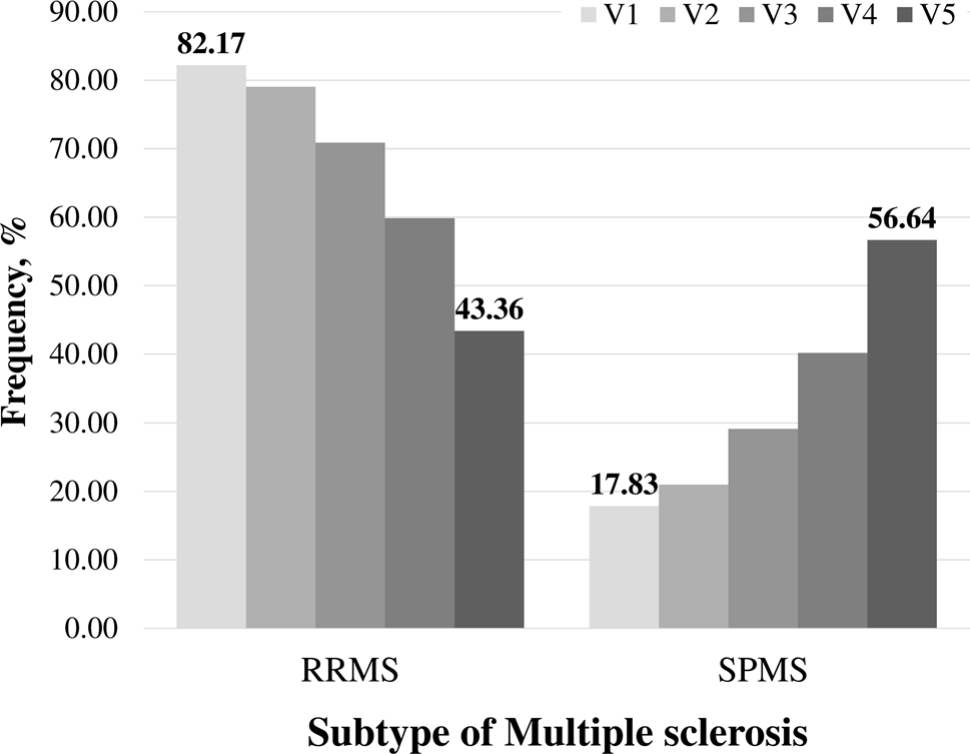
Distribution of stages of disease progression relapsing–remitting (RRMS) and secondary-progressive (SPMS) in the Multiple sclerosis cohort by visits at five-time points: V1—1st visit; V2—Visit after one or two years from the 1st visit; V3—Visit after 5 ± 1-year from 1st visit; V4—Visit after 10 ± 2 years from 1st visit; V5—Visit after 15—20 years from 1st visit

MS clinical courses in patients’ diseases cohort were distributed by five visits and considered the rate of disease progression (EDSS). Figure 2 shows the distribution of disease courses in the MS cohort by visits: in about 15–20 years, about half (56.6%) of the MS patients are with the SPMS subtype.

**Fig. 3:**
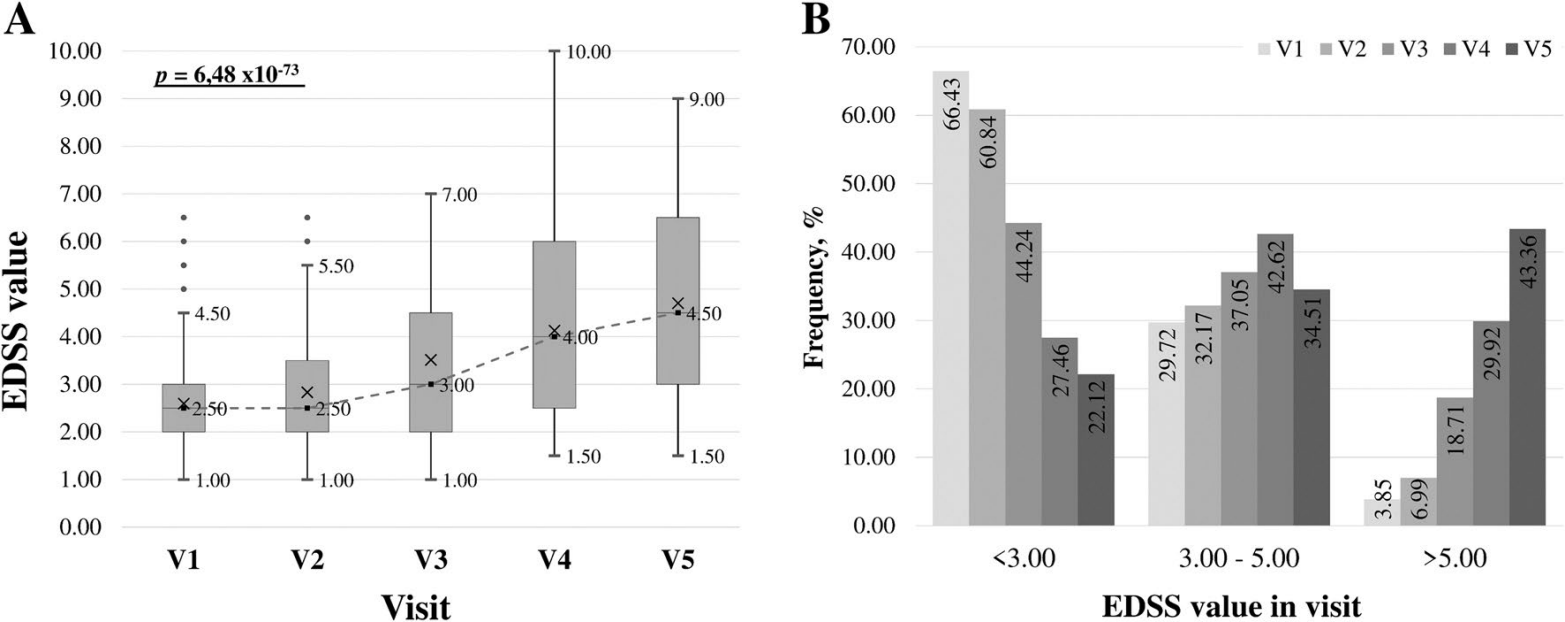
Distribution of stages of disease progression in the Multiple sclerosis cohort by visits at five-time points: V1—1st visit; V2—Visit after one or two years from 1st visit; V3—Visit after 5 ± 1-year from 1st visit; V4—Visit after 10 ± 2 years from 1st visit; V5—Visit after 15—20 years from 1st visit, by (**A**) increase in EDSS across five visits and (**B**) distribution of patients across visits by EDSS value. *p* in (**A**) statistical signification of Friedman test

The average values of the EDSS level were determined in the patient cohort stratified by gender at five-time points (by visits) (Table S1). Considering, that the EDSS data is not normally distributed, the statistical results are presented by medians and interquartile intervals (Fig. 3, Table S1).

To determine the time dynamics of the disease progression in the Latvian MS cohort, the studied collection was also divided into groups according to EDSS level: up to 3.00; from 3.00 to 5.00; 5.00 and above) at each visit; it can be seen (Table S2) that there is a statistically significant variation among patient EDSS values at these time points. A statistically significant difference (*p* = 6.48 × 10^–7^, Friedman Test) between all periods among patients was revealed (Fig. 3A).

Over time, the number of patients in the group with a low EDSS value (< 3.00) decreases, and those with a high EDSS value (> 5.00) increase. The proportion of patients in the middle group (EDSS value 3.00–5.00) remained at a similar frequency throughout the disease (Fig. 3B).

**Fig. 4:**
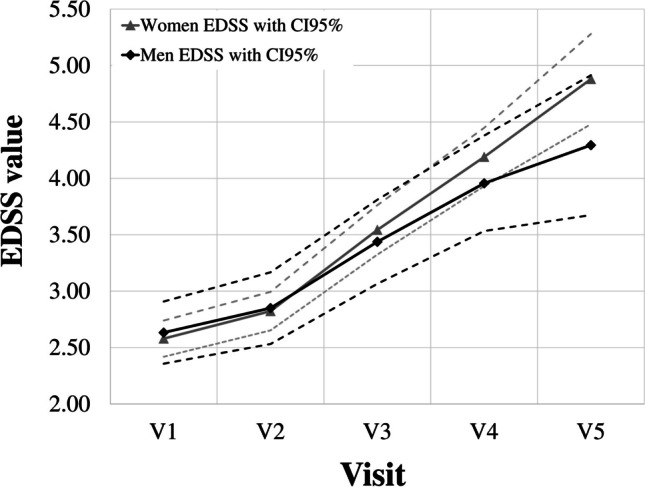
The result of a comparative analysis of the differences in average EDSS value between the genders at five study time points: V1—1st visit; V2—Visit after one or two years from the 1st visit; V3—Visit after 5 ± 1-year from 1st visit; V4—Visit after 10 ± 2 years from 1st visit; V5—Visit after 15—20 years from 1st visit, over a period of 15–20 years. Gray continuous and dashed black lines represent increased EDSS values in females and males, dashed lines—95% confidence intervals (CI 95%) of the specific groups

No difference in average EDSS value was found at each follow-up visit associated with disease progression between the genders (Fig. 4, Table 5). According to the results obtained, gender does not statically affect the EDSS value over time in the Latvian population, however, the EDSS value tended to increase more in women over a period of 15–20 years (Fig. 4).

**Table 5:** EDSS statistical analysis between the genders at different visits

Periods	Statistical analysis between gender groups	
	*p*	*η*
1st visit > A visit in a year or two from 1st visit (V1 > V2)	0.90	0.02
1st visit > Visit after 5 ± 1 years from 1st visit (V1 > V3)	0.54	0.08
1st visit > Visit after 10 ± 2 years from 1st visit (V1 > V4)	0.67	0.00
1st visit > Visit after 15—20 years from 1st visit (V1 > V5)	2.04 × 10^–2^	0.16
Visit after 15—20 years from 1st visit > Visit after 5 ± 1 years from 1st visit (V2 > V3)	0.39	0.08
Visit after 5 ± 1 years from 1st visit > Visit after 10 ± 2 years from 1st visit (V3 > V4)	0.41	0.06
Visit after 10 ± 2 years from 1st visit > Visit after 15—20 years from 1st visit (V4 > V5)	0.81	0.04

When comparing EDSS values between genders at different periods (among visits), only a statistically significant difference over a period of 15–20 years (from 1 visit > visit after 15–20 years, Mann–Whitney test) between groups of male and female patients was determined (Table 5, Fig. 4).

### EDSS value at the first visit related to the first symptoms and/or the age of the first visit

Considering that various primary symptoms of the disease affect the quality of life to a greater or lesser extent and this factor can influence the level of EDSS already during the first visit, we analysed the relationship of the EDSS value at the first visit with the presence of the first symptoms of the disease. In total, seven (7; Table 2) primary symptoms were identified for consideration in the experimental group of Latvian patients. Considering that one symptomatic group (memory or cognitive functions) was present only in two cases, this group was not included in the statistical analysis.

The mean EDSS values of the other patient groups at the first visit were not normally distributed, such that the comparison was based on the median values. The EDSS value (median) at the first visit is in the range of 2.00—2.50. The results obtained did not show a statistically significant difference between the comparison groups, although the p-value was close to the statistical limit (*p* = *0.078*, Table 6). When comparing the average EDSS values, it turned out that the highest first average EDSS value is presented in the Movement disorders group, and the lowest in the Sensory disturbances group of the first symptoms.

**Table 6:** Data on the association of the EDSS level at the first visit with the first symptoms of the disease

The first symptoms	EDSS value								Statistics analysis	
	Mean	SD	Min	Max	CI95%		Median	IQR	*p*	*η*
Sensory disturbances	**2.37**	0.86	1.00	5.00	2.14	2.60	2.50	1.00	0.078	0.15
Visual impairment	2.49	1.30	1.00	6.50	2.18	2.79	2.00	1.50		
Brain stem symptoms	2.42	1.25	1.00	6.00	1.96	2.88	2.00	2.00		
Cerebral symptoms	2.59	1.26	1.50	6.00	2.09	3.09	2.00	2.00		
Motor deficit	**2.91**	1.33	1.00	6.50	2.53	3.29	2.50	1.50		
Polysymptomatic onset	2.77	1.13	1.00	6.00	2.42	3.11	2.50	1.50		

*SD* standard deviation; *CI 95%* 95% confidential interval of the mean; *IQR* interquartile range

*p* Statistical signification of Kruskal–Wallis tests by median

Considering that the growth rate of EDSS value may be related to the age of the patient at the time of treatment and/or diagnosis, the present study also set the following objectives for testing: Is there a statistically significant relationship between the level of EDSS and its changes (or delta) at the visit and the patient's age at the first visit?

The correlation between patient age at the first visit and EDSS values is statistically significant (Table 7 A) and moderately close ( ≥|0.45|). Thus, it can be assumed that older patients are more likely to be diagnosed with a higher EDSS than younger patients. This correlation decreases with the increasing duration of illness (with each subsequent visit).

**Table 7:** Spearman's correlation between the patient’s age at the first visit and (A) the EDSS value at each visit or (B) ∆EDSS value in between visits

A		EDSS value in a visit						
	Visit:	V1	V2		V3	V4		V5
Spearman's correlation	*ρ*	0.46	0.46		0.46	0.45		0.31
	*p*	4.75 × 10^–16^	2.78 × 10^–16^		3.64 × 10^–16^	3.06 × 10^–13^		7.46 × 10^–4^
B		∆EDSS in between visits						
	The period between visits:	V1 > V2	V2 > V3	V3 > V4	V4 > V5	V1 > V3	V1 > V4	V1 > V5
Spearman's correlation	*ρ*	0.11	0.13	0.06	0.16	0.19	0.19	-0.03
	*p*	0.06	3.34 × 10^–2^	0.33	0.10	1.53 × 10^–3^	3.24 × 10^–3^	0.71

V1—1st visit; V2—Visit after one- or two-years from 1st visit; V3—Visit after 5 ± 1 year from 1st visit; V4—Visit after 10 ± 2 years from 1st visit; V5—Visit after 15–20 years from 1st visit

The correlation between the patient's age at the 1st visit and the change in EDSS (∆EDSS) between visits (Table 7 B) was not determined to be statistically significant because the level of correlation is very weak (*ρ* <*|0.40 |*).

Patient age is statistically significantly associated with EDSS size at the first visit, which accordingly affects its size at subsequent visits, but age is not associated with an increase in EDSS in the Latvian MS patient group.

### Use of medication without classifying medication into groups

The following question taken into consideration is whether the initiation of medicinal therapy at the 1st visit affects the EDSS changes (∆EDSS) over time, regardless of the duration of the therapy. Accordingly, the ∆EDSS between different two visits and the use of medication at the first visit were analysed (Table S3).

According to our data analysis, EDSS delta (∆EDSS) is not statistically significantly different between groups of patients with and without drug therapy. However, the V2 > V3 and V1 > V3 phase ∆EDSS between groups are close to the statistical significance. It is noteworthy that in all periods between visits (except V3 > V4), the increase in ∆EDSS was, on average, higher in patients with drug therapy compared to the group of patients without it (Table S3).

## Discussion 

The first part of our study describes the epidemiological and demographic data on multiple sclerosis in Latvia. There is an increase in the prevalence of MS in the region of the capital of the republic (about 60%) and large cities, and this trend is explained by factors associated with health services that may affect the diagnosis of MS; readily available compared to rural areas modern diagnostic methods (such as magnetic resonance imaging (MRI) for the public. The high proportion of non-smokers or quitters (about 80%) among patients with multiple sclerosis (MS) may be due to increased awareness of the harmful effects of smoking on health, including its association with multiple sclerosis [16], also because about half of the cohort of patients (47.64%) have higher education. Improved educational campaigns, health promotion efforts, and access to information are also contributing to this trend in Latvia.

In the collection of 288 MS patients, 204 (71%) are women, which corresponds to an average gender ratio of 2.4:1, and this is consistent with most European studies conducted to date [17–19]. Among the Baltic countries, our study also confirmed the predominance of women in MS among Latvians; in Lithuania, females were affected from 1.5 to 2 times more often than males [20]. Some possible factors that could contribute to this observed difference include variations in genetic susceptibility among populations, differences in healthcare systems and access to diagnosis, and potential variations in lifestyle or environmental factors.

In our study, no significant differences were found between genders regarding the presence of predominant symptoms at disease onset, however, the number of cases of the disease in the family history of females turned out to be almost 2 times more predominant concerning the male group (13.7% vs. 5.7% of cases). According to a meta-analysis of the worldwide prevalence of familial MS, the ratio of family history in affected females and males was 15.4% /13.7%, respectively [21]. Thus, the number of cases of a family history of MS in Latvian males was about three times lower than that found in other regional studies.

In the first year after disease onset, only one exacerbation was reported in 74.6% of patients from the general group. In the remaining cases, with a prevalence in the group of males almost twice (21.4% vs 11.27%, respectively) no incident was observed. Second exacerbation was registered in half of the cohort (48.7%) within a period of up to one year.

The most common symptom at presentation in our study was found to be optic manifestations (25.18%), followed by sensory disturbances (20.21%) and motor deficit (18%), which prevailed in the male group of patients to the female (23% vs 16%). In this study, we have only focused on the predominant symptom at diagnosis, although multiple symptoms were experienced by the patients. Interestingly, optic symptoms (37.8%) were the most common predominant symptom at diagnosis in Saudi Arabia and were found to be similar to our results [22]. However, according to the data presented in the Atlas of MS [19] the most common initial symptom of MS was sensory loss (40%), as well as motor disorders (39%) as the first clinical manifestation. In some of the available reports evaluating the cohort of Polish MS patients, the first clinical manifestation was usually motor deficit, followed by sensory symptoms and optic neuritis [17]. A study by Brola et al. reports the onset of the disease was also usually monosymptomatic (78.4%) and had the form of motor deficit (34.2%), optic neuritis (25.2%), and sensory disturbances (18.3%) of patients [23]. Therefore, the above distribution of the number and nature of the first symptoms turned out to be slightly different from our results, which may indicate a different initial course of the disease in different populations.

In our study, the nature of the first symptoms of MS reflected the level of EDSS, which characterizes the degree of progression of the disease, already at the first visit; the highest first mean EDSS value was assigned to the motor deficit group (2.91) and the lowest to the sensory impairment group (2.37) of the first symptoms. The first clinical manifestation in the form of a motor deficit was associated with a faster conversion to SPMS (*p* < *0.001*) in Rzepiński et al., [17] study, while the disease onset in the form of optic neuritis was associated with later conversion to SPMS (*p* = *0.002*). In addition, mobility is the most affected function in MS, previously published results have confirmed that mobility is lower in progressive types of MS [23].

In our study cohort, the mean age at the disease onset was 29.40 ± 9.42 years, while the minimum and maximum age of onset of symptoms were seven (7) and 54 years, respectively. These data corresponded with the data presented by Kułakowska et al. (30.4 ± 9.8 years) and Brola et al. (30.8 ± 9.8 years) and were lower than in the group of Termelett et al. (32.4 ± 10.3 years) Debouverie et al. (33 ± 10 years), and Jerković et al. (32.3 ± 10.9 years) [23–26].

In the case of the Latvian population, no differences were found in the mean age of the disease onset between the genders, however, according to other sources, this parameter was found in such correlation. According to Toncev et al., females in the Serbian district Sumadija were found to be significantly younger than males at disease onset (*p* = *0.023*, [27]).

It is assumed that in the natural course of the disease, the transition from RRMS to SPMS occurs between 5.8 and 19.1 years from the onset of symptoms, with the most likely median for this time being 19 years [28–30]. According to our data, the period of the RRMS stage before the transition to the SPMS stage was approximately the same between the genders and was calculated as 13.35 ± 9.05 years with a significant proportion (36.92%) progressing 16 or more years after the onset of the first symptoms. Our data are in good agreement with the results reported by Rzepinski et al., (12.7 ± 7.4 years), obtained in Potemkowski’s group (11.3 ± 4.2 years), for patients in the group of Eriksson et al., (12 ± 1.8 years) [17, 30, 31], and slightly different from the results obtained by Sand et al., (16.7 ± 2.0) years [32].

When considering the dynamics of the main clinical characteristics related to multiple sclerosis in the population of Latvia, their changes in the time from the beginning of the first visit to 20–25 years were studied through visits at five main time points. In the investigated group, the distribution of individual clinical multiple sclerosis variants at the first visit was in the ratio RRMS – 82%, SPMS – 18%, and corresponded to the generally accepted pattern for the MS course [19]. In a period of 20–25 years after the first visit (the start of disease registration), the disease variants were found to be distributed in the ratio: RRMS – 43.4%, SPMS – 56,8%, (Fig. 2). Thus, during the above period, about half of MS patients switched from RRMS to SPMS. There was wide variation in the estimated prevalence of SPMS within and across countries and in the proportion of patients with relapsing SPMS. This may be because of differences in SPMS definition, study design, or study duration, which should be explored in future studies.

In the Latvian disease cohort, the approximate age of transition to the SPMS stage was 42.98 ± 10.55 years and approximately the same between the genders (42.24 ± 10.73 years for females and 44.65 ± 10.07 years for males). Thus, the mean age of onset of SPMS progression in the Latvian MS population was lower compared to the age of patients in the study by Tutunku et.al, who found that 62% of patients with RRMS progressed to SPMS by age 75, with a median age at the onset of progression of 45 years [33]. According to the British Columbia Multiple Sclerosis Database, the median time to SPMS is 21 years and the median age of onset is 54 years [34].

To determine the temporal dynamics of disease progression in the Latvian MS cohort, the collection studied was also divided into three groups according to the level of EDSS; a statistically significant difference (*p* < *0.00001*) was found between all periods (visits) among patients. Thus, it can be concluded that LV diseases cohort cannot maintain a constant EDSS value over time or limit disease in patients with MS. (Fig. 2A); it is also obvious that there are groups of patients with varying degrees of disease progression: over time, at each follow-up visit, the number of patients in the group with low EDSS value (< 3.00), and those with high EDSS value (> 5.00), but there is also a part of patients in the group (with average EDSS 3.00–5.00), which remains at a similar frequency for 20–25 years (Fig. 3B). It can be assumed that these differences may be due to individual features in disease severity at diagnosis, genetic predisposition, environmental influences, access to health care and treatment, adherence to treatment, and lifestyle factors such as diet and exercise [35, 36]. In the Latvian study, gender was not a significant influencing factor on the rate of disease progression over short periods; a slight prevalence of progression rate (*p* < *0.05*) was observed in females compared to males only for a period of 15–20 years from the onset of illness (Table 5, Fig. 4). According to Rzepi´nski et al., faster conversion to SPMS was associated with the male gender in the Polish population [17]; male relapse-onset patients accumulate disability faster than female patients in the Ribbons et al. group [35]. Therefore, the above data turned out to be different from our results, which may indicate a different initial MS course among populations.

In the present study, we also tested for an association between the rate of disease progression and patient age at the time of treatment and/or diagnosis in our cohort of patients (Table 7); according to our data patient age was statistically significantly associated with EDSS value at the first visit, which accordingly affects its size at subsequent visits. Numerous studies support both age-associated cumulative neurodegeneration and unique CNS pathology as the underlying initiators of progression [36]. Older age at onset and longer MS duration are the most significant risk factors associated with progressive disease [34, 37]. Similarly, older age has been negatively associated with lower immune cell activity, along with lower gene expression and recruitment of progenitor oligodendrocytes and differentiation as well as subsequent consequences of reduced repair, remyelination, and other functions necessary to stabilize the relapsing–remitting course of the disease [38]. Thus, the data obtained by us supplement and confirm the significant influence of age-related physiological changes on the course of the disease and the accumulation of disability.

In the past decades, special attention has been paid to the effects of immune-modulating drugs (IMD) in MS patients. In our study, at the first visit, 96% of clinical cases showed clinical disease activity with magnetic resonance activity present in half of the cases (47%). At this time, a line of immunomodulatory and immunosuppressive drug therapy was initiated in almost a third cohort of patients. When considering the progression dynamics of the disease, we did not notice a statistically significant between groups of patients with and without drug therapy in the period from the beginning of therapy to 20–25 years. The obtained results do not give grounds for conclusions about the effectiveness of IMD since the compared groups of treated and untreated patients in our study were not homogeneous in terms of incidence, clinical, and radiological activity.

Several previous studies describing the natural course of multiple sclerosis have found a certain percentage of patients treated with immunosuppressive or immunomodulatory drugs [6, 39]. Both studies excluded a significant effect of the applied treatment on the assessed parameters, since the time of its application was too short, or there was no effect on the increase in disability. In the Polish cohort of RRMS patients, there was no influence of treatment with IMDs on the time to reach 4 and 6 points on the EDSS scale [17]. In this context, our study can also be viewed as an analysis of the natural history of multiple sclerosis in a cohort of Latvian patients.

An interesting fact, however, was the presence of a difference in ∆EDSS at the margin of statistical significance (*p* = *0.06*), between these groups after a period of approximately 5–9 years after the start of therapy. In addition, in the cases of all periods among visits (except V3 > V4), ∆EDSS was on average higher in patients with special drug therapy than in patients without it. This situation in the Latvian population can be explained by a certain difference in the degree of disease progression at the first visit, as well as the presence of resistance to treatment in certain patients: if patients develop resistance to the drug or have persistent disease activity despite treatment, their disease may progress with greater speed.

Our results emphasize the importance of early clinical MS parameters in determining the clinical disease variant and the time to conversion from RRMS to SPMS as well as predicting the rate of disability accrual in patients. In the age of general availability of disease-modifying therapy, their analysis is of particular importance for identifying patients requiring more aggressive treatment, as well as for a selection of proper treatment. In the present study, data on the long-term outcomes of disability in patients with MS in the Latvian population remain overwhelmingly consistent with the results obtained in other regions of the world.

The main limitation of this study was its retrospective nature. Another limitation could be the ratio of patients receiving IMDs, (about 32% in the first visit, as mentioned above), and the choice of therapy by the treating physician (non-randomized study).

Despite the limitations of our sample, this study draws its strength from the fact that it suggests the demographic and medical characteristics of people with MS in Latvia. He offers a unique insight into gender differences in how multiple sclerosis manifests itself. These results may help to assess the prevalence and demographic characteristics of MS in the Baltic region and thus, are expected to stimulate additional research that may help us confirm, understand, and better explain the current findings and their implications for the treatment of multiple sclerosis.

## Conclusions

This project is a multiple sclerosis study providing baseline information on more than 18% of patients from a disease cohort in Latvia (Baltic region) and offering useful information on differences between men and women in the presentation of the disease. Further prospective studies are needed.

In our study cohort of 288 MS patients, gender distribution, age of disease onset, nature of first symptoms, and the distribution of clinical types of the disease were consistent with the globally accepted pattern for the multiple sclerosis course.

### Supplementary Information

Below is the link to the electronic supplementary material.

Supplementary file1 (DOCX 25 KB)

## Data Availability

The data that support the findings of this study are available on Zenodo 10.5281/zenodo.8119564 on reasonable request.
