# Supplementary material for: Clinical characteristics and dynamics of disability progression in a cohort of patients with multiple sclerosis in Latvians
Source: Neurol Sci. 2024 Feb 23;45(7):3347–58. doi: 10.1007/s10072-024-07404-z (PMC11176098; doi:10.1007/s10072-024-07404-z)
Supplement: Supplementary file 1 — Supplementary file1 (DOCX 25 KB) [file 10072_2024_7404_MOESM1_ESM.docx]

Journal of Neurology  Multiple Sclerosis & Therapeutic

**Clinical characteristics and dynamics of disability progression in a cohort of patients with multiple sclerosis in Latvians**

J. Kalnina, I. Trapina*, N. Sjakste, N. Paramonova

* Corresponding author: email: [ilva.trapina@lu.lv](mailto:ilva.trapina@lu.lv) (I.T.)

**Table S1** The time dynamics (by visit) of the disease progression of EDSS (*Expanded Disability Status Scale)* in the Latvian MS cohort.

| **Patients groups** | **Value of EDSS** | | | | | | | | **Statistical analysis^** |
| --- | --- | --- | --- | --- | --- | --- | --- | --- | --- |
|  | **Mean** | **SD** | **Min** | **Max** | **CI95%** | | **Median** | **IQR** | **p** |
| *1st visit* | | | | | | | | | |
| All | 2.59 | 1.19 | 1.00 | 6.50 | 2.46 | 2.73 | 2.50 | 1.13 | - |
| Female | 2.58 | 1.16 | 1.00 | 6.50 | 2.42 | 2.74 | 2.50 | 1.50 | 0.90 |
| Male | 2.63 | 1.26 | 1.00 | 6.50 | 2.36 | 2.91 | 2.00 | 1.50 |  |
| *A visit in a year or two* | | | | | | | | | |
| All | 2.83 | 1.30 | 1.00 | 6.50 | 2.68 | 2.98 | 2.50 | 1.50 | - |
| Female | 2.82 | 1.23 | 1.00 | 6.50 | 2.65 | 2.99 | 2.50 | 1.50 | 0.64 |
| Male | 2.85 | 1.46 | 1.00 | 6.50 | 2.53 | 3.17 | 2.50 | 1.50 |  |
| *Visit after 5 +/- 1 years* | | | | | | | | | |
| All | 3.51 | 1.59 | 1.00 | 7.00 | 3.32 | 3.70 | 3.00 | 2.50 | - |
| Female | 3.54 | 1.56 | 1.00 | 7.00 | 3.32 | 3.76 | 3.50 | 2.50 | 0.51 |
| Male | 3.44 | 1.67 | 1.00 | 7.00 | 3.07 | 3.81 | 2.50 | 2.50 |  |
| *Visit after 10 +/- 2 years (245)* | | | | | | | | | |
| All | 4.12 | 1.74 | 1.50 | 10.00 | 3.91 | 4.34 | 4.00 | 3.50 | - |
| Female | 4.19 | 1.74 | 1.50 | 10.00 | 3.93 | 4.45 | 4.00 | 3.50 | 0.36 |
| Male | 3.96 | 1.75 | 1.50 | 7.00 | 3.53 | 4.38 | 3.50 | 3.38 |  |
| *Visit after 15 - 20 years (113 = 79 + 34)* | | | | | | | | | |
| All | 4.70 | 1.79 | 1.50 | 9.00 | 4.37 | 5.04 | 4.50 | 3.50 | - |
| Female | 4.88 | 1.79 | 2.00 | 9.00 | 4.48 | 5.28 | 5.00 | 3.50 | 0.12 |
| Male | 4.29 | 1.78 | 1.50 | 7.50 | 3.67 | 4.91 | 4.00 | 3.13 |  |

SD – standard deviation; CI 95% - 95% confidential interval of mean; IQR - interquartile range.

^ - statistical analyze between gender groups; p - statistical signification of Mann Whitney tests;

**Table S2.** Dynamics of disease progression (change of EDSS) over time in the Latvian multiple sclerosis cohort.

| **∆EDSS group*** | **Sample** | | **Value of ∆EDSS between visits** | | | | | | | | | | | **Statistical analysis** | | | | |  |
| --- | --- | --- | --- | --- | --- | --- | --- | --- | --- | --- | --- | --- | --- | --- | --- | --- | --- | --- | --- |
|  | **N** | **%** | | **Mean** | **SD** | **Min** | **Max** | **CI95%** | | | **Median** | | **IQR** | | | **p** | | **η** | |
| *1st visit > A visit in a year or two form 1st visit (V1 > V2)* | | | | | | | | | | | | | | | | | | |  |
| <3.00 | 190 | 66.43 | | 0.21 | 0.46 | 0.00 | 3.00 | 0.14 | 0.27 | 0.00 | | 0.13 | | | 3.11  x10^-2^ | | 0.03 | | |
| 3.00 - 5.00 | 85 | 29.72 | | **0.33** | 0.60 | 0.00 | 2.50 | 0.20 | 0.46 | 0.00 | | 0.50 | | |  |  |  |  |  |
| >5.00 | 11 | 3.85 | | 0.00 | 0.00 | 0.00 | 0.00 | 0.00 | 0.00 | 0.00 | | 0.00 | | |  |  |  |  |  |
| *1st visit > Visit after 5 +/- 1 years form 1st visit (V1 > V3)* | | | | | | | | | | | | | | | | | | |  |
| <3.00 | 183 | 65.83 | | 0.84 | 1.05 | 0.00 | 5.00 | 0.69 | 0.99 | 0.50 | | 1.00 | | | 1.72  x10^-4^ | | 0.03 | | |
| 3.00 - 5.00 | 84 | 30.22 | | **1.13** | 0.92 | 0.00 | 3.00 | 0.93 | 1.33 | 1.00 | | 1.38 | | |  |  |  |  |  |
| >5.00 | 11 | 3.96 | | 0.23 | 0.26 | 0.00 | 0.50 | 0.05 | 0.40 | 0.00 | | 0.50 | | |  |  |  |  |  |
| *1st visit > Visit after 10 +/- 2 years form 1st visit (V1 > V4)* | | | | | | | | | | | | | | | | | | |  |
| <3.00 | 161 | 66.26 | | 1.45 | 1.26 | 0.00 | 5.50 | 1.25 | 1.65 | 1.00 | | 1.50 | | | 1.03  x10^-4^ | | 0.03 | | |
| 3.00 - 5.00 | 73 | 30.04 | | 1.86 | 1.30 | 0.00 | 7.00 | 1.55 | 2.16 | 1.50 | | 1.50 | | |  |  |  |  |  |
| >5.00 | 9 | 3.70 | | 0.39 | 0.42 | 0.00 | 1.00 | 0.07 | 0.71 | 0.50 | | 0.75 | | |  |  |  |  |  |
| *1st visit > Visit after 15 - 20 years form 1st visit (V1 > V5)* | | | | | | | | | | | | | | | | | | |  |
| <3.00 | 68 | 60.71 | | 2.07 | 1.57 | 0.00 | 5.50 | 1.69 | 2.45 | 1.50 | | 3.00 | | | 0.08 | | 0.10 | | |
| 3.00 - 5.00 | 37 | 33.04 | | 2.00 | 1.26 | 0.00 | 5.00 | 1.58 | 2.42 | 2.00 | | 2.00 | | |  |  |  |  |  |
| >5.00 | 7 | 6.25 | | 0.79 | 0.64 | 0.00 | 1.50 | 0.20 | 1.37 | 1.00 | | 1.50 | | |  |  |  |  |  |
| *Visit after 15 - 20 years form 1st visit > Visit after 5 +/- 1 years form 1st visit (V2 > V3)* | | | | | | | | | | | | | | | | | | |  |
| <3.00 | 169 | 60.79 | | 0.60 | 0.86 | 0.00 | 5.00 | 0.47 | 0.73 | 0.50 | | 1.00 | | | 6.00  x10^-3^ | | 0.05 | | |
| 3.00 - 5.00 | 89 | 32.01 | | 0.90 | 0.95 | 0.00 | 3.50 | 0.70 | 1.10 | 0.50 | | 1.50 | | |  |  |  |  |  |
| >5.00 | 20 | 7.19 | | 0.30 | 0.34 | 0.00 | 1.00 | 0.14 | 0.46 | 0.25 | | 0.50 | | |  |  |  |  |  |
| *Visit after 5 +/- 1 years form 1st visit > Visit after 10 +/-2 years form 1st visit (V3 > V4)* | | | | | | | | | | | | | | | | | | |  |
| <3.00 | 108 | 44.44 | | 0.62 | 0.75 | 0.00 | 3.00 | 0.48 | 0.76 | 0.50 | | 1.00 | | | 2.60  x10^-2^ | | 0.18 | | |
| 3.00 - 5.00 | 94 | 38.68 | | **0.88** | 1.12 | 0.00 | 6.00 | 0.65 | 1.11 | 0.50 | | 1.50 | | |  |  |  |  |  |
| >5.00 | 41 | 16.87 | | 0.34 | 0.39 | 0.00 | 1.50 | 0.22 | 0.47 | 0.50 | | 0.50 | | |  |  |  |  |  |
| *Visit after 10 +/- 2 years form 1st visit > Visit after 15 - 20 years form 1st visit (V4 > V5)* | | | | | | | | | | | | | | | | | | |  |
| <3.00 | 33 | 29.20 | | 0.52 | 0.79 | 0.00 | 2.50 | 0.24 | 0.79 | 0.00 | | 0.50 | | | 4.61  x10^-2^ | | 0.12 | | |
| 3.00 - 5.00 | 46 | 40.71 | | **0.93** | 1.05 | 0.00 | 4.50 | 0.62 | 1.25 | 0.50 | | 1.50 | | |  |  |  |  |  |
| >5.00 | 34 | 30.09 | | 0.41 | 0.43 | 0.00 | 1.50 | 0.26 | 0.56 | 0.50 | | 0.63 | | |  |  |  |  |  |

* EDSS values were divided into three groups: (1) <3.00; (2) 3.00 to 5.00 and (3) >5.00; to compare the dynamics of disease progression for each visit by different levels of MS.

N – Number of patients; % - frequency of distributions; In bold – the highest value of ∆EDSS in time period.

SD – standard deviation; CI 95% - 95% confidential interval of mean; IQR - interquartile range.

p - Statistical signification of Kruskal–Wallis tests.

**Table S3.** Dynamics of disease progression by visits depending on the start of therapy in the Latvian multiple sclerosis cohort.

| **Therapy at the 1st visit** | **Patient group** | | ∆EDSS | | | | | | | | **Statistics analysis** | |
| --- | --- | --- | --- | --- | --- | --- | --- | --- | --- | --- | --- | --- |
|  | **N** | **%** | **Mean** | **SD** | **Min** | **Max** | **CI95%** | | **Median** | **SKI** | **p** | η |
| *1st visit > A visit in a year or two form 1st visit (V1 > V2)* | | | | | | | | | | | | |
| No | 195 | 68.18 | 0.22 | 0.46 | 0.00 | 2.50 | 0.15 | 0.28 | 0.00 | 0.50 | 0.42 | 0.06 |
| Yes | 91 | 31.82 | **0.28** | 0.58 | 0.00 | 3.00 | 0.16 | 0.40 | 0.00 | 0.50 |  |  |
| *1st visit > Visit after 5 +/- 1 years form 1st visit (V1 > V3) (1^st^ five year period)* | | | | | | | | | | | | |
| No | 191 | 68.71 | 0.86 | 1.03 | 0.00 | 5.00 | 0.71 | 1.00 | 0.50 | 1.00 | *0.07* | 0.09 |
| Yes | 87 | 31.29 | **1.01** | 0.96 | 0.00 | 3.50 | 0.81 | 1.22 | 0.50 | 1.00 |  |  |
| *1st visit > Visit after 10 +/- 2 years form 1st visit (V1 > V4)* | | | | | | | | | | | | |
| No | 175 | 72.02 | 1.52 | 1.24 | 0.00 | 6.50 | 1.33 | 1.71 | 1.50 | 1.50 | 0.20 | 0.07 |
| Yes | 68 | 27.98 | **1.57** | 1.39 | 0.00 | 7.00 | 1.23 | 1.90 | 1.00 | 2.00 |  |  |
| *1st visit > Visit after 15 - 20 years form 1st visit (V1 > V5)* | | | | | | | | | | | | |
| No | 103 | 91.96 | 1.94 | 1.42 | 0.00 | 5.50 | 1.66 | 2.21 | 1.50 | 2.00 | 0.32 | 0.04 |
| Yes | 9 | 8.04 | **2.28** | 1.92 | 0.00 | 5.50 | 0.80 | 3.76 | 2.50 | 3.25 |  |  |
| *Visit after 15 - 20 years form 1st visit > Visit after 5 +/- 1 years form 1st visit (V2 > V3)* | | | | | | | | | | | | |
| No | 127 | 45.68 | 0.59 | 0.83 | 0.00 | 3.50 | 0.44 | 0.74 | 0.50 | 1.00 | *0.06* | 0.07 |
| Yes | 151 | 54.32 | **0.74** | 0.92 | 0.00 | 5.00 | 0.59 | 0.89 | 0.50 | 1.00 |  |  |
| *Visit after 5 +/- 1 years form 1st visit > Visit after 10 +/-2 years form 1st visit (V3 > V4) (second five-year period)* | | | | | | | | | | | | |
| No | 81 | 33.33 | **0.77** | 0.96 | 0.00 | 6.00 | 0.55 | 0.98 | 0.50 | 1.25 | 0.94 | 0.02 |
| Yes | 162 | 66.67 | 0.63 | 0.86 | 0.00 | 6.00 | 0.49 | 0.76 | 0.50 | 1.00 |  |  |
| *Visit after 10 +/- 2 years form 1st visit > Visit after 15 - 20 years form 1st visit (V4 > V5) (third five-year period)* | | | | | | | | | | | | |
| No | 38 | 33.63 | 0.61 | 0.95 | 0.00 | 4.50 | 0.29 | 0.92 | 0.00 | 1.00 | 0.74 | 0.06 |
| Yes | 75 | 66.37 | **0.68** | 0.81 | 0.00 | 3.50 | 0.49 | 0.87 | 0.50 | 1.00 |  |  |

N – Number of patients; % - frequency of distributions; In bold – the highest value of ∆EDSS in time period.

SD – standard deviation; CI 95% - 95% confidential interval of mean; IQR - interquartile range.

p - Statistical signification of Kruskal–Wallis tests.
